# Impact of COVID-19 on the hospitalization, treatment, and outcomes of intracerebral and subarachnoid hemorrhage in the United States

**DOI:** 10.1371/journal.pone.0248728

**Published:** 2021-04-14

**Authors:** Vijay M. Ravindra, Ramesh Grandhi, Alen Delic, Samuel Hohmann, Ernie Shippey, David Tirschwell, Jennifer A. Frontera, Shadi Yaghi, Jennifer J. Majersik, Mohammad Anadani, Adam de Havenon

**Affiliations:** 1 Department of Neurosurgery, Naval Medical Center San Diego, San Diego, California, United States of America; 2 Department of Neurosurgery, Clinical Neurosciences Center, University of Utah, Salt Lake City, Utah, United States of America; 3 Department of Neurology, Clinical Neurosciences Center, University of Utah, Salt Lake City, Utah, United States of America; 4 Research Analytics, Vizient Inc., Irving, Texas, United States of America; 5 Department of Neurology, University of Washington, Seattle, Washington, United States of America; 6 Department of Neurology, NYU Langone Health, New York, New York, United States of America; 7 Department of Neurology, Brown University, Providence, Rhode Island, United States of America; 8 Department of Neurology, Washington University in St. Louis, St. Louis, Missouri, United States of America; University of Ioannina School of Medicine, GREECE

## Abstract

**Objective:**

To examine the outcomes of adult patients with spontaneous intracranial and subarachnoid hemorrhage diagnosed with comorbid COVID-19 infection in a large, geographically diverse cohort.

**Methods:**

We performed a retrospective analysis using the Vizient Clinical Data Base. We separately compared two cohorts of patients with COVID-19 admitted April 1–October 31, 2020—patients with intracerebral hemorrhage (ICH) and those with subarachnoid hemorrhage (SAH)—with control patients with ICH or SAH who did not have COVID-19 admitted at the same hospitals in 2019. The primary outcome was in-hospital death. Favorable discharge and length of hospital and intensive-care stay were the secondary outcomes. We fit multivariate mixed-effects logistic regression models to our outcomes.

**Results:**

There were 559 ICH-COVID patients and 23,378 ICH controls from 194 hospitals. In the ICH-COVID cohort versus controls, there was a significantly higher proportion of Hispanic patients (24.5% vs. 8.9%), Black patients (23.3% vs. 20.9%), nonsmokers (11.5% vs. 3.2%), obesity (31.3% vs. 13.5%), and diabetes (43.4% vs. 28.5%), and patients had a longer hospital stay (21.6 vs. 10.5 days), a longer intensive-care stay (16.5 vs. 6.0 days), and a higher in-hospital death rate (46.5% vs. 18.0%). Patients with ICH-COVID had an adjusted odds ratio (aOR) of 2.43 [1.96–3.00] for the outcome of death and an aOR of 0.55 [0.44–0.68] for favorable discharge. There were 212 SAH-COVID patients and 5,029 controls from 119 hospitals. The hospital (26.9 vs. 13.4 days) and intensive-care (21.9 vs. 9.6 days) length of stays and in-hospital death rate (42.9% vs. 14.8%) were higher in the SAH-COVID cohort compared with controls. Patients with SAH-COVID had an aOR of 1.81 [1.26–2.59] for an outcome of death and an aOR of 0.54 [0.37–0.78] for favorable discharge.

**Conclusions:**

Patients with spontaneous ICH or SAH and comorbid COVID infection were more likely to be a racial or ethnic minority, diabetic, and obese and to have higher rates of death and longer hospital length of stay when compared with controls.

## Introduction

The novel coronavirus severe acute respiratory syndrome-CoV-2 (SARS-CoV-2) has led to a global pandemic of patients with COVID-19, the disease caused by the coronavirus, with significant impact [1]. Neurological complications of COVID-19, including encephalopathy, seizure, and cerebrovascular disease, have been described [2–4]; a recent meta-analysis demonstrated that SARS-CoV-2 increased risk of ischemic and cryptogenic stroke [5]. Increased use of anticoagulants in the context of COVID-19–associated thrombotic events, endothelial injury that may predispose vessels to rupture, and COVID-19–related cerebral sinus thrombosis may predispose COVID-19 patients to higher rates of intracranial hemorrhage. The clinical outcomes of patients with intracranial hemorrhage with COVID-19 are not fully known, with only small cohorts of patients with intracranial hemorrhage and comorbid COVID-19 published to date [6–13]. The objective of the current study is to examine the outcomes of adult patients with spontaneous intracerebral hemorrhage (ICH) and those with nontraumatic subarachnoid hemorrhage (SAH) who were diagnosed with comorbid COVID-19 in a national dataset. We hypothesize that concomitant COVID-19 will increase mortality in this population. We also predict that patients with COVID-19 will have a longer length of stay in the intensive care unit (ICU) and in the hospital.

## Methods

We performed a retrospective analysis using the Vizient Clinical Data Base, a healthcare analytics platform employed by 568 participating US hospitals for the purpose of benchmarking clinical performance, costs, and outcomes [14]. Institutional review board approval was not required to use this deidentified data set. We identified one cohort of adult patients who were hospitalized between April 1 and October 31, 2020, with International Classification of Diseases, 10^th^ revision (ICD-10) codes for ICH (I61*) as a primary or secondary discharge diagnosis and excluded those with ICD-10 coding for SAH (I60*) or ischemic stroke (I63*, H34.1). We identified a second cohort of adult patients who were hospitalized during the same period with SAH who did not have ICH or ischemic stroke. Patients with elective admission or receiving hospice care at admission were excluded. We identified patients who had comorbid COVID-19, determined by the ICD-10 code U07.1, which is reserved for laboratory-confirmed infection with SARS-CoV-2. We compared the patients with COVID-19 in each group to similar groups (ICH or SAH) without COVID-19 treated at the same hospitals in 2019.

The primary outcome was in-hospital death. Favorable discharge, defined as discharge home or to acute rehabilitation, was a secondary outcome. Conversely, unfavorable discharge was defined as discharge to skilled nursing facility or death. We also evaluated length of hospital and ICU stay. We report descriptive statistics and test for differences using the chi-squared test, Student’s t-test, or Wilcoxon rank sum tests. We stratified our cohorts by US Census region and hospital size (≤150 beds, 151–250 beds, 251–500 beds, and ≥500 beds) to investigate for differences in our outcomes based on the stratifications. To account for patient clustering by hospital, we fit mixed-effects logistic regression models to our outcomes. The mixed-effects model estimates a separate intercept for each hospital to account for between-hospital differences, such as case volume or proportion of cases with COVID, thus treating each hospital as its own sample in the statistical analysis. The models were adjusted for patient age, sex, race/ethnicity, Elixhauser comorbidity score, intubation, acute coronary syndrome, acute renal failure, pulmonary embolism, diabetes, congestive heart failure, obesity, and smoking. In a sensitivity analysis, we restricted the cohort to patients who had a diagnosis of ICH or SAH that was present on hospital admission. For all analyses, significance was set at p≤0.05 and analysis was performed using Stata 16.0 (StataCorp, College Park, TX).

## Results

### ICH-COVID

For the ICH cohort, we included 559 ICH-COVID patients and 23,379 controls from 194 hospitals, of which 56 were in the Northeast Census region, 47 in the Midwest, 63 in the South, and 28 in the West. The control cohort was comprised of mostly white patients (57.1%), whereas in the ICH-COVID cohort there were higher proportions of Hispanic patients (24.5% vs. 8.9%), Asian patients (6.1% vs. 4.7%), and Black patients (23.3% vs. 20.9) (Table 1). There were also significantly higher proportions of patients with obesity, diabetes, atrial fibrillation, or congestive heart failure, more male patients, and more patients <75 years in the ICH-COVID cohort (Table 1). There was a lower proportion of smokers in the ICH-COVID cohort. In the control cohort, the ICH was present on admission in 93.0% of patients, it was present on admission in only 53.5% of the ICH-COVID cohort (p<0.001). This suggests that almost half of ICH in patients with COVID-19 happened during the hospitalization. A significantly higher proportion of patients in the ICH-COVID cohort were administered heparin, enoxaparin, and dalteparin during hospitalization (69.8% vs. 55.4%, p<0.001). In the ICH-COVID patients, the proportion who received an anticoagulant (heparin, enoxaparin, or dalteparin) was higher in patients who did not have ICH present on admission than those who did (83.5% vs. 57.9%, p<0.001). In patients with ICH and a documented NIHSS, there were 93 ICH-COVID patients and 6,865 controls, with a median (IQR) NIHSS of 15 (5–24) versus 9 (3–19) (p<0.001).

**Table 1:** Baseline demographics and outcomes of patients with intracerebral hemorrhage with or without COVID-19 infection.

Variable	Control	ICH-COVID	p value
	(2019)	(April–October 2020)	
	(n = 23,378)	(n = 559)	
**Age category in years**			<0.001
**<18**	383 (1.6%)	suppressed*	
**18–50**	4,224 (18.1%)	120 (21.5%)	
**51–64**	6,547 (28.0%)	200 (35.8%)	
**65–74**	5,399 (23.1%)	126 (22.5%)	
**75–79**	2,434 (10.4%)	41 (7.3%)	
**≥80**	4,391 (18.8%)	68 (12.2%)	
**Age <75 years**	16,553 (70.8%)	450 (80.5%)	<0.001
**Male sex**	12,794 (54.7%)	345 (61.7%)	0.001
**Race/Ethnicity**^**a**^			<0.001
**White**	13,350 (57.1%)	171 (30.6%)	
**Black**	4,893 (20.9%)	130 (23.3%)	
**Hispanic**	2,081 (8.9%)	137 (24.5%)	
**Asian**	1,090 (4.7%)	34 (6.1%)	
**Other/Unknown**	1,964 (8.4%)	87 (15.6%)	
**ICH diagnosis present on admission**	21,738 (93.0%)	299 (53.5%)	<0.001
**Elixhauser score**			
**Median, IQR**	3, 2–5	4, 3–5	<0.001
**Mean±SD**	3.3±2.0	4.2±2.3	<0.001
**Obese**	3,145 (13.5%)	174 (31.1%)	<0.001
**Smoker**	2,696 (11.5%)	18 (3.2%)	<0.001
**Atrial fibrillation**	4,474 (19.1%)	132 (23.6%)	0.008
**Hypertension**	15,854 (67.8%)	354 (63.3%)	0.025
**Diabetes**	6,657 (28.5%)	242 (43.3%)	<0.001
**Dyslipidemia**	9,628 (41.2%)	235 (42.0%)	0.685
**Congestive heart failure**	3,334 (14.3%)	107 (19.1%)	0.001
**Interfacility transfer**	10,450 (44.7%)	245 (43.8%)	0.228
**Seen in emergency department (n = 23,289)**	15,588 (68.5%)	339 (63.7%)	0.019
**Intubated**	7,038 (30.1%)	358 (64.0%)	<0.001
**Administered heparin, enoxaparin, or dalteparin during hospitalization**	12,945 (55.4%)	390 (69.8%)	<0.001
**Acute coronary syndrome**	1,195 (5.1%)	79 (14.1%)	<0.001
**Acute renal failure**	4,854 (20.8%)	308 (55.1%)	<0.001
**Pulmonary embolus**	598 (2.6%)	39 (7.0%)	<0.001
**Venous sinus or deep vein thrombosis**	467 (2.0%)	10 (1.8%)	0.727
**Length of hospital stay in days (mean±SD)**	10.5±15.9	21.6±21.0	<0.001
**Length of ICU stay in days**^**b**^ **(mean±SD)**	6.0±9.4	16.5±16.7	<0.001
**Favorable discharge**	12,040 (51.5)	146 (26.1)	<0.001
**In-hospital death**	4,216 (18.0)	260 (46.5)	<0.001

Binary variables presented as n, %; ordinal variables as median, IQR; interval variables as mean (SD). P values calculated with the chi-squared test for binary variables, the Wilcoxon rank sum test for ordinal variables, and Student’s t-test for interval variables.

*Value suppressed for low count.

^a^White and Black racial categories are non-Hispanic.

^b^Length of ICU stay restricted to patients with >24 hours spent in intensive care.

**Fig 1:**
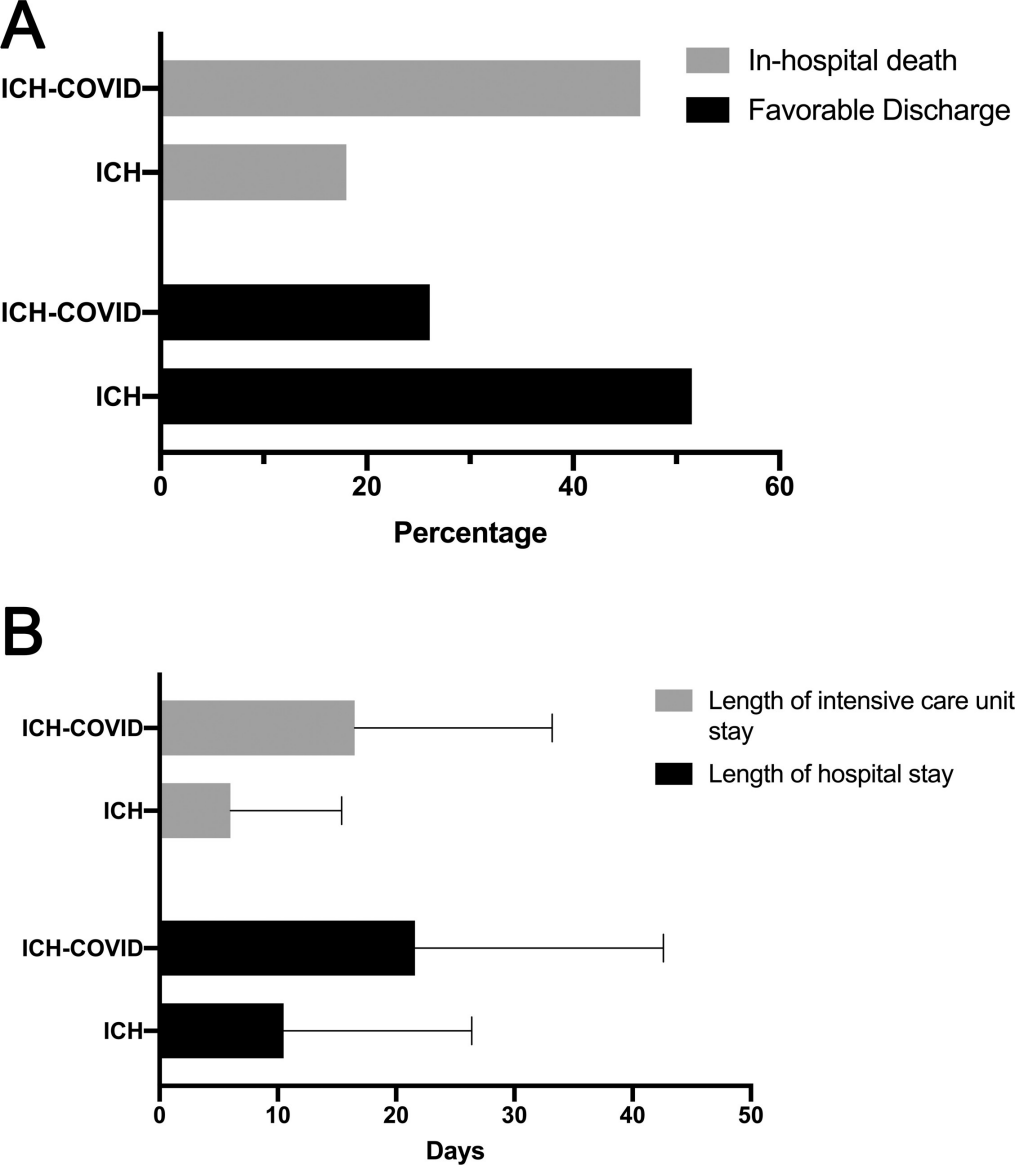
(A) Bar graph demonstrating rates of in-hospital death and favorable discharge among ICH-COVID and ICH cohorts. (B) Bar graph demonstrates rates of length of ICU and hospital stay among ICH-COVID and ICH cohorts.

Additionally, a significantly higher proportion of the ICH-COVID patients were intubated (64.0% vs. 30.1%) or experienced acute coronary syndrome (14.1% vs. 5.1%), acute renal failure (55.1% vs. 20.8%), or pulmonary embolus (7.0% vs. 2.6%) during hospitalization. No differences were observed in the rates of venous sinus or deep vein thrombosis. In the ICH-COVID cohort, there was a longer hospital stay (21.6 days vs. 10.5 days, p<0.001) and longer ICU stay (16.5 days vs. 6.0 days, p<0.001) (Fig 1), lower rate of favorable discharge (26.1% vs. 51.5%, p<0.001), and higher rate of in-hospital death (46.5% vs. 18.0%, p<0.001). We did not observe differences in our primary or secondary outcomes in the stratifications by Census region (p>0.4 for in-hospital death and favorable discharge) or hospital size (p>0.2 for both outcomes). The mixed-effects logistic regression model demonstrated that patients with ICH-COVID had an adjusted odds ratio (aOR) of 2.64 [95% CI, 2.01–3.47] for the outcome of death and an aOR of 0.55 [95% CI, 0.41–0.74] for favorable discharge (Table 2). These associations remained true in the sensitivity analysis where we only included patients who had ICH that was present on hospital admission (aOR 2.01 and 0.69, respectively; p<0.001 for both).

**Table 2:** Mixed-effects logistic regression fit to in-hospital death and favorable discharge, showing odds ratios for ICH patients with comorbid COVID-19 compared with controls.

	Death				Favorable discharge			
	Odds ratio	95% CI	SE	p value	Odds ratio	95% CI	SE	p value
**Unadjusted**	4.10	3.45–4.87	0.36	<0.001	0.32	0.27–0.37	0.03	<0.001
Adjusted*	2.43	1.96–3.00	0.26	<0.001	0.55	0.44–0.68	0.06	<0.001

* Adjusted for patient age, sex, race/ethnicity, Elixhauser comorbidity score, intubation, acute coronary syndrome, acute renal failure, pulmonary embolism, diabetes congestive heart failure, obesity, and smoking.

### SAH-COVID

For the SAH cohort, we included 212 SAH-COVID patients and 5,029 controls from 119 hospitals, of which 35 were in the Northeast Census region, 25 in the Midwest, 40 in the South and 19 in the West. A majority of the control group were white (52.4%), whereas in the SAH-COVID cohort the proportion of white patients was 29.3%, because of larger Hispanic and Black proportions (Table 3). More patients in the SAH-COVID cohort had obesity, atrial fibrillation, diabetes, dyslipidemia, and congestive heart failure comorbidities than in the control group, and fewer were smokers (Table 3). SAH was not present on admission in over half of patients with COVID-19. No significant difference in the proportion of patients who were administered heparin, enoxaparin, or dalteparin during hospitalization was observed. In the 212 SAH-COVID patients, the proportion who received an anticoagulant was higher in patients who did not have SAH present on admission than those who did (80.6% vs. 65.4%, p<0.001).

**Table 3:** Baseline demographics and outcomes of patients with subarachnoid hemorrhage.

Variable	Control	SAH-COVID	p value
	(2019)	(April–July 2020)	
	(n = 5,029)	(n = 212)	
**Age category in years**			0.793
**<18**	74 (1.5%)	suppressed*	
**18–50**	1,471 (29.3%)	58 (27.4%)	
**51–64**	1,704 (33.9%)	78 (36.8%)	
**65–74**	996 (19.8%)	41 (19.3%)	
**75–79**	305 (6.1%)	16 (7.8%)	
**≥80**	479 (9.5%)	17 (8.0%)	
**Age <75 years**	4,245 (84.4%)	179 (84.4%)	0.993
**Male sex**	2,127 (42.3%)	107 (50.5%)	0.018
**Race/Ethnicity**^**a**^			<0.001
**White**	2,637 (52.4%)	62 (29.3%)	
**Black**	910 (18.1%)	44 (20.8%)	
**Hispanic**	645 (12.8%)	63 (29.7%)	
**Asian**	198 (3.9%)	suppressed	
**Other/Unknown**	639 (12.7%)	34 (16.0%)	
**SAH diagnosis present on admission**	4,633 (92.1%)	104 (49.1%)	<0.001
**Elixhauser score**			
**Median, IQR**	2, 1–4	4, 2–5	<0.001
**Mean±SD**	2.8±2.1	3.8±2.2	<0.001
**Obese**	740 (14.7%)	66 (31.1%)	<0.001
**Smoker**	835 (16.6%)	13 (6.1%)	<0.001
**Atrial fibrillation**	625 (12.4%)	35 (16.5%)	0.079
**Hypertension**	3,214 (63.9%)	122 (57.6%)	0.059
**Diabetes**	1,015 (20.2%)	99 (46.7%)	<0.001
**Dyslipidemia**	1,684 (33.5%)	93 (43.9%)	0.002
**Congestive heart failure**	634 (12.6%)	48 (22.6%)	<0.001
**Interfacility transfer**	2,895 (57.6%)	86 (40.6%)	<0.001
**Seen in emergency department (n = 5,016)**	2,869 (59.7%)	135 (65.5%)	0.091
**Intubated**	1,591 (31.6%)	144 (67.9%)	<0.001
**Administered heparin, enoxaparin, or dalteparin during hospitalization**	3,472 (69.0%)	155 (73.1%)	0.208
**Acute coronary syndrome**	325 (6.5%)	31 (14.6%)	<0.001
**Acute renal failure**	917 (18.2%)	117 (55.2%)	<0.001
**Pulmonary embolus**	96 (1.9%)	23 (10.9%)	<0.001
**Venous sinus or deep vein thrombosis**	102 (2.0%)	suppressed	0.421
**Length of hospital stay in days (mean±SD)**	13.4±15.8	26.9±23.4	<0.001
Length of ICU stay in days (mean±SD)*	9.6±9.8	21.9±19.0	<0.001
**Favorable discharge**	3,292 (65.5%)	88 (31.1%)	<0.001
**In-hospital death**	744 (14.8%)	91 (42.9%)	<0.001

Binary variables presented as n, %; ordinal variables as median, IQR; interval variables as mean (SD). P values calculated with the chi-squared test for binary variables, the Wilcoxon rank sum test for ordinal variables, and Student’s t-test for interval variables.

*Value suppressed for low count.

^a^White and Black racial categories are non-Hispanic.

^b^Length of ICU stay restricted to patients with >24 hours spent in intensive care.

**Fig 2:**
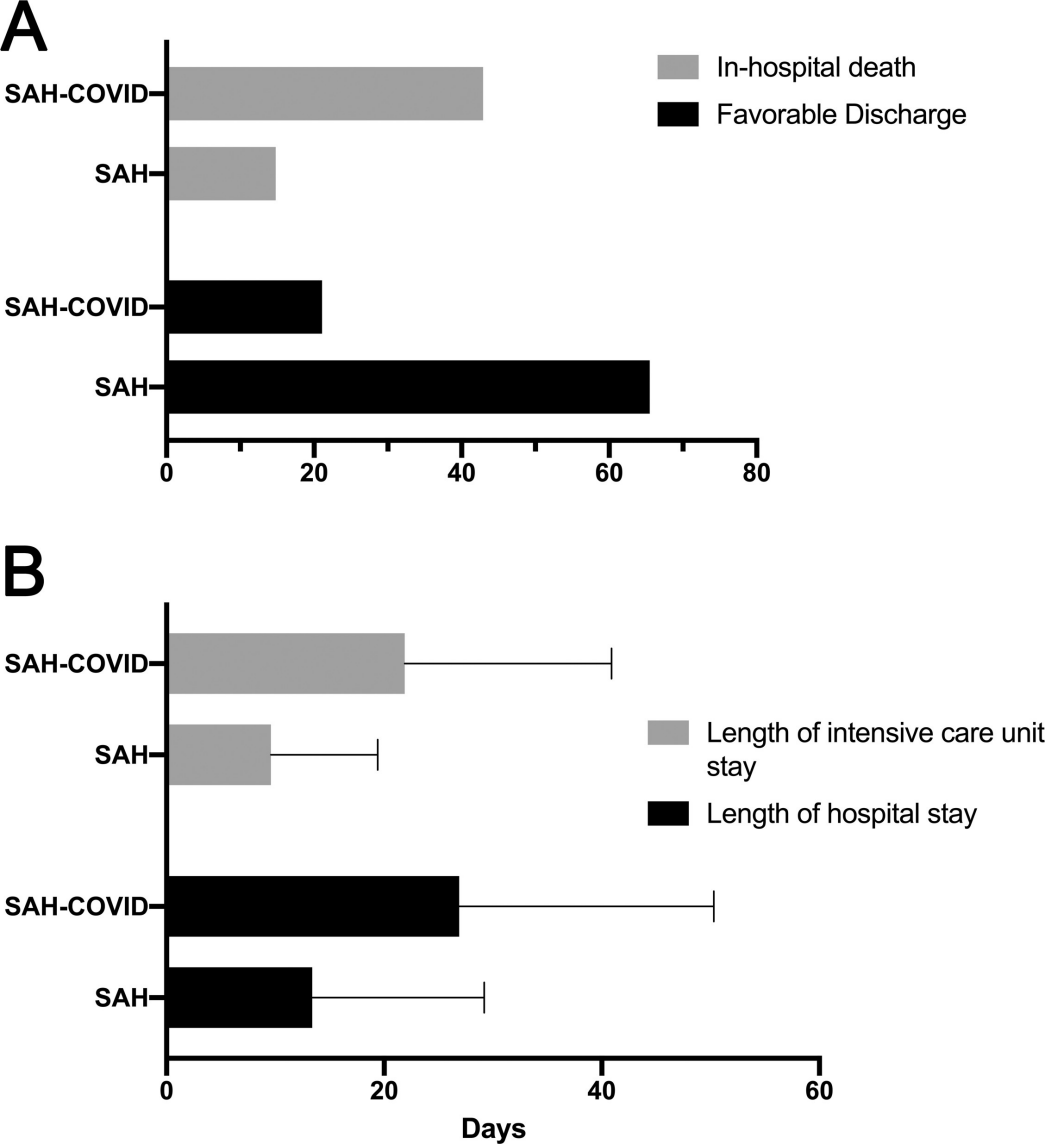
(A) Bar graph demonstrating rates of in hopital death and favorable discharge among SAH-COVID and SAH cohorts. (B) Bar graph demonstrates rates of length of ICU and hospital stay among SAH-COVID and SAH cohorts.

Similarly, significantly higher proportions of patients in the SAH-COVID cohort were intubated and experienced acute coronary syndrome, acute renal failure, and pulmonary embolus. No differences were observed among rates of venous sinus or deep vein thrombosis. In the SAH-COVID cohort, there was a longer hospital stay (26.9 vs. 13.4 days, p<0.001), longer ICU stay (21.9 vs. 9.6 days, p<0.001), lower rate of favorable discharge (31.1% vs. 65.5%, p<0.001), and more in-hospital death (42.9% vs. 14.8%, p<0.001) (Fig 2). In patients with SAH and a documented NIHSS, there were 15 SAH-COVID patients and 851 controls, with a median (IQR) NIHSS of 13 (7–26) versus 3 (0–14) (p<0.001). We did not find differences in outcome rates by hospital size, but there was a higher rate of favorable discharge in SAH-COVID patients in the West and South than Northeast or Midwest (Northeast: 21.6%, Midwest 25.6%, South: 45.3%, West: 31.4%, p = 0.022). The mixed-effects logistic regression model demonstrated that patients with SAH-COVID had an aOR of 1.81 [1.26–2.59] for the outcome of death and an aOR of 0.54 [0.37–0.78] for favorable discharge (Table 4). In the sensitivity analysis where we only included patients with SAH present on admission, the association between SAH-COVID and in-hospital mortality remained significant (aOR 2.11, 1.22–3.65), but was not for the favorable discharge (aOR 0.71, 0.44–1.15).

**Table 4:** Mixed-effects logistic regression fit to in-hospital death and favorable discharge, showing odds ratios for SAH patients with comorbid COVID-19 compared with controls.

	Death				Favorable discharge			
	Odds ratio	95% CI	SE	p value	Odds ratio	95% CI	SE	p value
**Unadjusted**	4.34	3.26–5.78	0.64	<0.001	0.25	0.18–0.33	0.04	<0.001
Adjusted*	1.81	1.26–32.59	0.33	0.001	0.54	0.37–0.78	0.10	0.001

* Adjusted for patient age, sex, race/ethnicity, Elixhauser comorbidity score, intubation, acute coronary syndrome, acute renal failure, pulmonary embolism, diabetes congestive heart failure, obesity, and smoking.

## Discussion

ICH and SAH in patients with comorbid COVID-19 infection are associated with a higher rate of in-hospital death and a lower rate of favorable discharge when compared with control patients without COVID-19. We also found that half of ICH and SAH in COVID-19 patients occurred after the patients had been admitted to the hospital and were more likely in patients who had received anticoagulation. This information could be useful as emerging therapies for COVID-19 are developed and warrant consideration for use in this at-risk population. Using a national dataset, we have estimated the impact of concomitant COVID-19 infection by assessing the resultant outcomes in the setting of ICH and SAH. Initially during the COVID-19 pandemic, there was a decrease in the incidence of hospitalization for acute SAH across multiple countries [15]; however, outcomes for those admitted with SAH with and without COVID-19 during this period are lacking. Importantly, the neurological manifestations of COVID-19 continue to emerge. The most common manifestation is toxic metabolic encephalopathy, while the most commonly reported stroke type thus far has been ischemic, which is thought to be associated with the overall hypercoagulable state [16, 17]. In a multinational study on hospitalized patients with SARS-CoV-2 infection, Shahjouei et al. [18] demonstrated an overall stroke risk of 0.5% (pooled 0.9%) with ischemic heart disease (OR 2.5, p = 0.006) and mechanical ventilaton (OR 1.9, p = 0.03) as independent predictors. Although less common, a subset of neurologic complications has been thought to be due a cytokine storm and multisystem inflammation resulting in acute demyelination, vasculitis, necrotizing encephalopathy, and posterior reversible encephalopathy syndrome [19]. Tsivgoulis et al. [20] undertook a review of neurological manifestations during the COVID-19 pandemic and found predominantly mild neurological manifestations in the majority of infected patients, while multiple comorbidites were present with severe neurological disease.

In this study, we demonstrated that patients in the ICH-COVID cohort were significantly more likely to be Hispanic, Asian, or Black than patients in the control group, which mostly comprised white patients. The COVID-19 pandemic has placed a disproportionate burden of morbidity and mortality on the Black and Hispanic populations [21], which is similar to previous viral pandemics [22–24]. Parcha et al. [25] studied geographic variation and racial disparities in COVID-19 and demonstrated a threefold higher cumulative incidence rate (per 100,000) in Blacks compared with Whites, with a twofold higher crude mortality rate. In both the ICH-COVID and SAH-COVID comparisons, we found higher proportions of Blacks, Asians, and Hispanics than in the control groups. Douglas and Subica [21] demonstrated that a higher percentage of Blacks and Hispanics predicted more total COVID-19 tests per 1000 persons (p<0.05), but with lower predicted hospitalizations and ICU bed access. Their findings indicate that Blacks and Hispanics are not only at risk secondary to inequitable access to healthcare resources in disadvantaged communities but also that social disadvantage may drive inequities related to access to care. The predisposition for higher proportions of Blacks and Hispanics to be affected with COVID-19 is mirrored in this sample examining patients experiencing ICH or SAH, likely with similar socioeconomic driving forces. Further inquiry into this phenomenon is warranted and necessary.

A higher proportion of patients in the SAH-COVID and ICH-COVID cohorts had obesity, atrial fibrillation, diabetes, and congestive heart failure than in the control cohorts. Smati et al. [26] found that overweight status and obesity carried a poorer early prognosis in patients with type II diabetes and COVID-19, but the impact of obesity diminished in those greater than 75 years of age. Interestingly, in both the ICH-COVID and SAH-COVID cohorts in our study, there were lower proportions of patients who smoked versus controls. This may indicate that COVID-19–related morbidity substitutes as a pulmonary risk factor and creates an additive effect in the setting of spontaneous intracranial hemorrhage, either ICH or SAH, and a poorer outcome. This may also be a reflection of the virulence of COVID-19 to affect patients with presumably normal pulmonary function as a nonsmoker.

The strength of this study is that the Vizient Clinical Data Base is a large, validated database that comprises a sample of the population presenting to both community and teaching/academic hospitals in different regions of the country, making the findings more generalizable than single-center cohort studies [27, 28]. Administrative data from Vizient has previously been used to examine large cohorts of patients with stroke [29, 30]. On the other hand, there are also several limitations to the current investigation. The study cohort is derived from an administrative database, so data were queried using billing codes, which have varying accuracy. The Vizient database is primarily used by academic institutions, which could introduce bias and limit generalizability to community-based practices. We also used a historical cohort from 2019, which represents an adequate comparison, but may limit the generalizability. Additionally, coding for ICH and SAH may be mixed, despite different clinical implications for each. In an effort to overcome this limitation, we only included patients with unique codes of ICH or SAH. A total of 4,378 cases were excluded secondary to mixed coding; inclusion of these patients in the analysis did not change the overall results of the investigation, so mixed cases were censored to isolate the analysis to the conditions of interest. The severity of ICH (volume, intraventricular extension) and SAH were not available through this dataset, and severity of hemorrhage likely plays a role that was thus undetected in this investigation. Although our data suggest more severe ICH and SAH in patients with COVID based on NIHSS, the high potential for selection bias limits the conclusions that can be drawn from this finding.The use of anticoagulants was limited to identify heparin, enoxaparin, or dalteparin; other medications may have been missed via this method. We also do not have dosing information. With emerging evidence regarding outcomes and heterogeneity of COVID-19, it is challenging to generalize the results revealed from this investigation. Despite these limitations, we have demonstrated compelling evidence that concomitant COVID-19 infection significantly impacts outcomes in patients experiencing ICH or SAH.

## Conclusion

Patients experiencing spontaneous ICH or SAH with comorbid COVID-19 infection had worse outcomes with respect to discharge disposition and in-hospital death. Additionally, hospital and ICU length of stay were longer and comorbid events including pulmonary embolism, acute coronary syndrome, and respiratory failure were all higher in the COVID-19 cohort, as were race and ethnic minorities. Half of the patients with COVID-19 developed ICH or SAH during their hospitalization and were more likely to have received anticoagulation. This finding warrants further investigation to improve outcomes for these high-risk cerebrovascular patients.
